# Drug Design: Where We Are and Future Prospects

**DOI:** 10.3390/molecules26227061

**Published:** 2021-11-22

**Authors:** Giuseppe Zagotto, Marco Bortoli

**Affiliations:** 1 Department of Pharmaceutical Sciences, University of Padova, Via Marzolo 5, 35131 Padova, Italy; 2 Institute of Computational Chemistry and Catalysis (IQCC) and Department of Chemistry, Faculty of Sciences, University of Girona, C/M. A. Capmany 69, 17003 Girona, Spain; marco.bortoli@udg.edu

**Keywords:** drug discovery, precision medicine, pharmacodynamics, pharmacokinetics

## Abstract

Medicinal chemistry is facing new challenges in approaching precision medicine. Several powerful new tools or improvements of already used tools are now available to medicinal chemists to help in the process of drug discovery, from a hit molecule to a clinically used drug. Among the new tools, the possibility of considering folding intermediates or the catalytic process of a protein as a target for discovering new hits has emerged. In addition, machine learning is a new valuable approach helping medicinal chemists to discover new hits. Other abilities, ranging from the better understanding of the time evolution of biochemical processes to the comprehension of the biological meaning of the data originated from genetic analyses, are on their way to progress further in the drug discovery field toward improved patient care. In this sense, the new approaches to the delivery of drugs targeted to the central nervous system, together with the advancements in understanding the metabolic pathways for a growing number of drugs and relating them to the genetic characteristics of patients, constitute important progress in the field.

## 1. Introduction

The fascinating path of drug discovery shares many features with a very complex and multidimensional maze, in which the medicinal chemist starts from the chemical space, more than 10^60^ small drug-like molecules of which only about 10^8^ of have been synthesized so far [1], and has to find the way to the drug at the center of the maze. A further intriguing property of this maze is that the walls and the center keep moving with time. Obviously, the maze is the body, with its barriers and transporters, and the center is the target site for a drug, which we call a receptor in its extensive sense. Very often, for sake of simplicity, the properties of a drug are grouped in pharmacodynamics and pharmacokinetics, but it must be remembered that a drug is a single entity, comprising both groups. The pharmacodynamic and pharmacokinetic properties stem from the chemistry of the drug molecule; the chemistry of the body, which is also made up by molecules; and the chemistry of the water that is interacting with both. All these molecules are not fixed in place, but continuously move. Particularly important are the movements happening when a molecule interacts with the target or off-target sites, leading to a biological effect. The understanding of body–drug interactions is a very complex problem where the properties of the molecules, which the medicinal chemist can know either from fundamental chemistry rules or from empirical observations of complex macromolecules and the biology of living organisms, can help to find the right way to the drug. A substantial simplification that is often made by medicinal chemists is to forget about the time course: in other words, to consider both the small molecules and the macromolecules as fixed. In this short perspective on the drug discovery process, a static situation is considered first, then some considerations on how to possibly account for the chemical motions are made. Moreover, the discussion is limited mainly to chemical entities complying with Lipinski′s rule of five (Ro5), even if there are new trends looking at chemical entities beyond the Ro5 (bRo5) [2]. This enlargement of the chemical space from Ro5 to bRo5 molecules may reflect the enlargement of the target space, up to now often limited at the inhibition of enzymatic action antagonizing the action of signal transmission in the body, to the modulation of macromolecule behavior as it happens in protein–protein or protein–nucleic acid interaction. This enlargement of the target space also requires new tools to manage the absorption, distribution, metabolism, and excretion (ADME) and toxicity of the new active pharmaceutical ingredients. In this short perspective, the attention is focused only on active pharmaceutical ingredients and not on excipients, drug delivery, or dosing regimens. Furthermore, pathways leading to “me-too drugs”, even if very important for the pharmaceutical industry, are not considered.

## 2. The Lead Discovery

### 2.1. Target Selection and Validation: Possible Expansion of Chemical Space

When looking for a new drug, the first choice to be made, almost always, is the selection of the target disease [3]. This decision is based mostly on economic considerations but can also be made following the discovery of a new biochemical pathway leading to a pathological state, or the finding of an important biomarker. Sometimes, it is the observation of the properties of a substance or metabolite that sparks the interest to better investigate a topic. Recently, many pharmaceutical companies have been searching in universities or in contract research organizations (CROs) [4,5] for new biochemical pathways or new chemical entities to treat diseases. The selection of the target disease is a basic choice, since the overall failure rate in drug discovery is very high, over 96%, including a 90% failure rate during clinical development, and costs are massive [6]. Once the target disease is defined, the next step toward the discovery of a new drug is the selection and validation of the biological target: a protein [7], a nucleic acid [8], or a different biochemical structure critical to the development of the disease, that can be characterized and is druggable [2]. During the last thirty years, a tremendous effort has been devoted to the selection of the correct target, with the widespread use of genome-wide association studies [9]. Even if this approach is increasingly showing problems [10], it remains a fundamental investigation tool for target selection, and in the last several years, since the chemical space available has expanded with the massive introduction of monoclonal antibodies, there are many new opportunities, such as the challenging task of the delivery of monoclonal antibodies to the central nervous system.

### 2.2. From Hit to Lead: Structure-Guided Drug Design and Beyond

**Figure 1:**
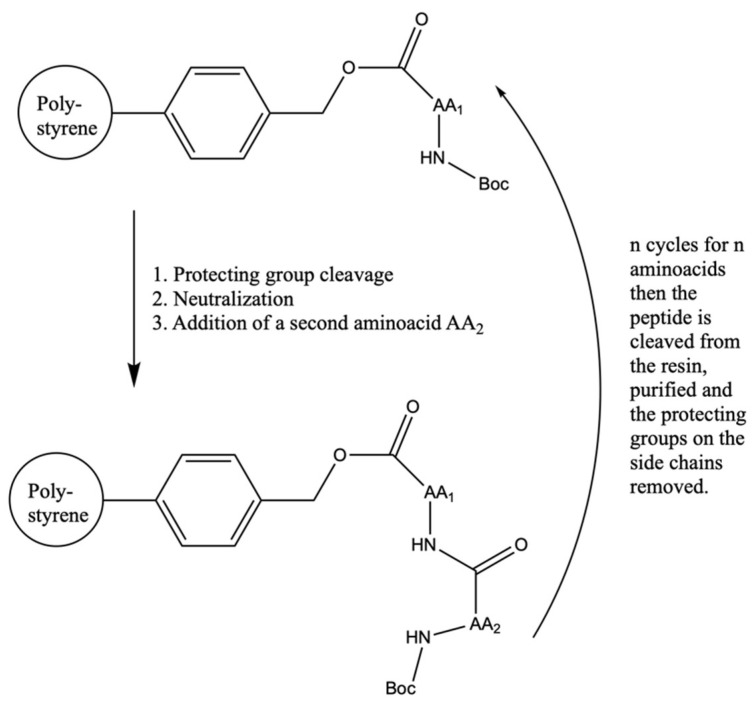
Merrifield peptide synthesis on a polystyrene support.

Once the target is selected and validated, there are many possible pathways leading to a chemical entity able to bind the target. The selection of chemical structures able to bind the selected target is usually conducted by screening large libraries of molecules, available in-house or from outsourcing, against the selected biological target [5]. The molecule collection may be made up of virtual or real chemical entities. Real chemical entities, until the second half of the last century, were obtained by a separate synthesis of every single molecule or by isolating them from natural sources. After Merrifield’s discovery of solid phase synthesis (Figure 1) [11], the development of computers able to manipulate large datasets, and the possibility of performing biochemical and biological experiments using very small amounts of compounds, combinatorial chemistry was introduced to help and speed up the drug discovery and optimization process [12].

**Figure 2:**
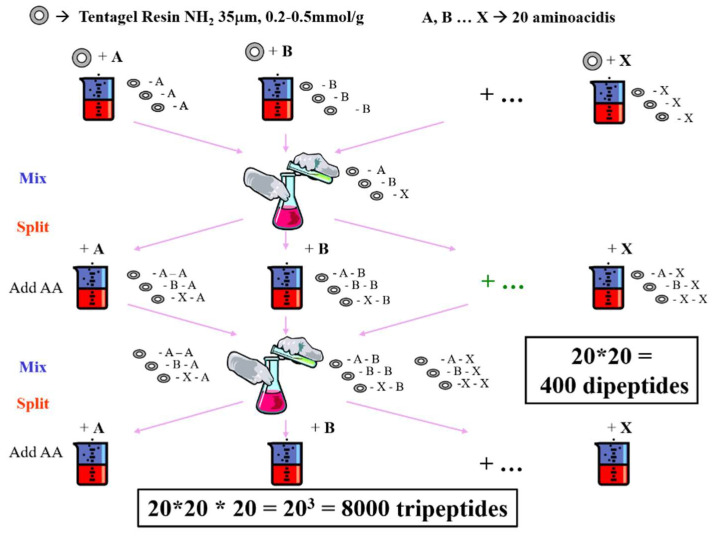
Schematic illustration of split–and–mix combinatorial synthesis.

Combinatorial chemistry started in the mid-1980s with the synthesis of hundreds of thousands of peptides on solid supports in parallel or with the split–and–mix methods (Figure 2). Lam et al. [13] introduced the one-bead, one-compound combinatorial peptide libraries, which first allowed only one bond to be formed: the peptidic amide bond. The following step in the evolution of solid phase synthesis was to make the phosphoroester bond connecting the nucleotides using mainly phosphoramidite chemistry [14,15]. In 1992, Bunin and Ellman reported the first example of a small-molecule combinatorial library [16] and started the era of the synthesis of libraries of small drug-like molecules satisfying the Ro5.

The combinatorial synthesis of drug-like compounds was strongly pushed on by the pharmaceutical industry around the turn of the century, and so were the analytical tools, with respect to the chemical characterization of very small quantities of substance, the decoding of chemical libraries, and the biochemical assays. The high-throughput era had begun, and someone referred to this as an accelerated evolution in the search of new active compounds: instead of using millions of years to select molecules able to protect life against predators or perform precise biochemical tasks, scientists could obtain the same result in a period that was very short in comparison. At the end of the last century, libraries of oligopeptides, oligonucleotides, and drug-like small molecules [17] were routinely prepared on automated synthesizers, providing pure substances in a rapid and efficient manner. However, the solid phase synthesis of oligosaccharide libraries was not yet feasible due to either the presence of functional groups of similar reactivity on each saccharide monomer, or to the fact that a new stereogenic center is created each time a glycosidic bond is formed [18]. Another consideration that slowed the development of the solid phase synthesis of oligosaccharide libraries was that while proteins and nucleic acids are genetically encoded structures, polysaccharides are not. This made polysaccharides ill-defined and unappealing targets for many investigators and pharmaceutical managers, as carbohydrates are considered particularly important only in few signal transduction pathways [19] and vaccines [20].

**Figure 3:**
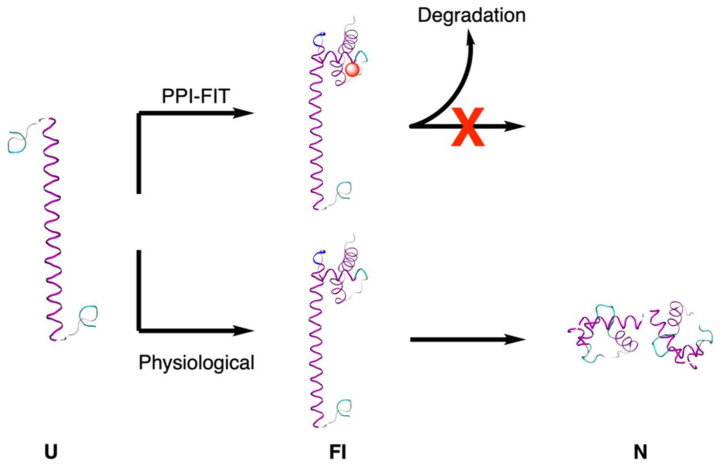
Schematic representation of the PPI-FIT approach to protein regulation. U = unfolded; FI = folding intermediate; N = native. The red sphere represents the drug molecule.

The availability of a very large number of compounds from combinatorial synthesis, in-house libraries, robotics, high-throughput screening methods, and fast structure determination constitutes a great help in the drug discovery process. Moreover, computers and software able to store, organize, and manage a huge, and continuously growing, amount of data are available to the pharmaceutical field. Despite this, we need something else to improve and speed up the pharmacodynamics in drug discovery when a validated target is established. No recipe is available for this, but taking into consideration the time evolution of chemical processes, instead of the static snapshots of the target structure as determined by X-ray crystallography, NMR, or cryo-electron microscopy, can help the medicinal chemist. To better understand the target structure behavior while it is performing its biological task, we must extrapolate the time course from many structure determinations, often crystallographic or NMR. The determination of the time course for a biochemical process, which is fascinating, although very challenging, will allow us to understand how the signal is managed by the validated target structures such as proteins, nucleic acids, or other biochemical players. More accurate structural data and improved chemistry software will allow a better look at the structure and its changes with time, environment, and regulator molecules. A recent example that explicitly considers the time evolution of a target molecule is the PPI-FIT method, which involves the targeting of intermediates along the path of protein folding (Figure 3) [21]. These structures are regarded as the druggable targets because they present binding pockets not present in the protein’s final structure. The drug-intermediate interaction should stabilize the complex, thus preventing the protein from reaching its native conformation. The method employs computer simulations together with experimental techniques, and supports the idea that folding intermediate targeting could represent a useful way to regulate protein levels. Regarding the crystallographic support to the drug-discovery process, it was recently reported that a detailed understanding of the interactions between drugs and their targets is crucial to developing the best possible therapeutic agents, and that structure-based drug design still relies on the availability of high-resolution structures obtained primarily through X-ray crystallography [22]. Working on a single crystal is marginally useful to understanding the enzyme movements during the catalytic process and to plan possible molecular structures interacting or interfering with different conformational states of the enzyme. At the moment, it is possible to combine different crystal snapshots to have an idea of the enzyme conformational changes during the catalytic process, as it was performed for the ubiquitous enzymes α-d-phosphohexomutases [23].

To characterize the various enzyme conformations involved in the isomerization of 1-phospho to 6-phosphohexoses, 15 high-resolution crystal structures of the phosphoglucomutase enzyme while performing the isomerization of glucose 1-phosphate to glucose 6-phosphate were obtained. Glucose 1,6-bisphosphate undergoes a 180° reorientation between the two phosphoryl transfer steps of the reaction. The enzyme with the phosphoserine bound to a Mg^2+^ ion has the same conformation at the beginning of the catalytic process, when it is bound to the substrate glucose 1-phosphate, and at the end of it, when it is bound to the product glucose 6-phosphate. During the reorientation of the sugar, when the catalytic serine is in the dephosphorylated state and bound to the glucose 1,6-bisphosphate intermediate, the enzyme has a different structure. In the future, the structure of such intermediates of the enzymes may suggest new drug molecules eventually able to trap, in these intermediate conformations, even the enzymes that are currently not druggable. It is also possible to use an in silico methodology combining a classic and quantum mechanics approach [24] to better understand the catalytic path, as is performed on the selenoenzyme glutathione peroxidase in the reduction reaction of hydrogen peroxides and organic hydroperoxides by glutathione. NMR [25] and EPR [26] measurements can also feed data to molecular in silico calculations to determine the evolution of a protein with time, although limited to the active site or oligonucleotide structures. At the moment, dealing with the changes of structures with time in protein–protein interaction, as in GPCR receptors and the intracellular effector proteins or in a protein regulator [27], or with protein-oligonucleotides binding, as in transcription regulators, is much more complicated, but very appealing [28].

### 2.3. Speeding up Screening and Design: Artificial Intelligence in Drug Discovery

In addition, artificial intelligence (AI) is finding its way in helping the process of speeding up drug discovery [29] with the design of improved experiments and more sophisticated machine learning (ML) algorithms to better understand the behavior of the target structure when performing its biological task. The increasing volume of available data has given a strong impulse to computer-aided drug design, with the latest developments focused on the applications of deep learning (DL) [30,31]. These methods take advantage of the already known concept of artificial neural networks and, due to the augmented performance of calculators, increase their complexity, reaching a much improved performance compared to other ML algorithms [32,33,34]. Moreover, their application reaches to not only the molecular discovery process of drug design (as in structure-activity predictions [35] or de novo design [36]), but also the synthetic (or retrosynthetic) route [37,38] and formulation design [39,40,41], and takes steps to also encompass fields that, while still pertaining to the drug discovery process, lie outside of wet laboratory activity, such as product quality assurance, marketing, and clinical trial management [29,30]. Other recent studies that benchmarked DL against other machine learning algorithms for properties prediction, using large biomolecular datasets comprising hundreds of thousands of compounds, consistently showed that deep neural networks are the best performing approach [42,43]. In addition to properties prediction or screening, DL has been employed in de novo design. As an example, a particular neural network was designed with the aim of transforming a set of molecular structures of known properties into a continuous representation of a molecular structure that could be exploited to maximize a desired property, and then reversibly transformed into an optimized molecular structure expressing such desired property [44]. With this machinery, novel structures were proposed that showed potential specific anticancer properties [45] and a predicted activity against dopamine receptor type 2 [46]. Analogous approaches employing the power of DL have been used to develop tools for the design of a molecule that can adapt best to a given 3D protein pocket [47] or that can display a particular desired property [48]. Moreover, DL has been integrated with more traditional computer techniques to decrease the computational cost without losing their predictive power. For instance, a DL-driven quantum mechanical approach was employed to efficiently calculate electronic wavefunctions of possible drug candidates [49], and the application of a neural network trained on MD simulations showed that the calculation of free energies of transfer of 1500 small molecules is possible with small errors [50]. Finally, DL techniques can also complement the drug discovery process, shedding light on the fundamental interactions that take place in the human body at a molecular level and on their disruption at the onset of disease. On the other hand, the lack of a large amount of high-quality data, required to train the algorithms successfully, is one of the main drawbacks of these methods. For example, the atomistic structure of many proteins, which is essential to understand their mechanism of action, is still not known. Again, DL has proven to be effective in these areas, as demonstrated by the successful development of the AlphaFold method [51] and its extension, ColabFold [52], two of the most promising structure prediction algorithms that, starting from an amino acid sequence, can predict the 3D folded structure of a protein with an accuracy competing with experimental structures [51]. Another feature that renders the obtained data sometimes hard to interpret but, more importantly, provides no insight into the underlying biochemical mechanism, is the fact that DL algorithms operate as a black box [35,53]. Nevertheless, the clear knowledge of the molecular cause of a pathological condition combined with the ability to obtain through AI-driven methods an effective and efficient compound without severe side effects in a very short time can impart a strong impulse to successful drug development. Moreover, as these techniques continue to develop, treatment possibilities increase, opening new possible choices to fight pathological conditions. Again, DL has proven useful in aiding the fine tailoring of the best treatment choice based on the analysis of patient data such as life history, previous diagnostics, and manifested symptoms [53].

### 2.4. One Size Does Not Fit All: From General to Precision Medicine

**Figure 4:**
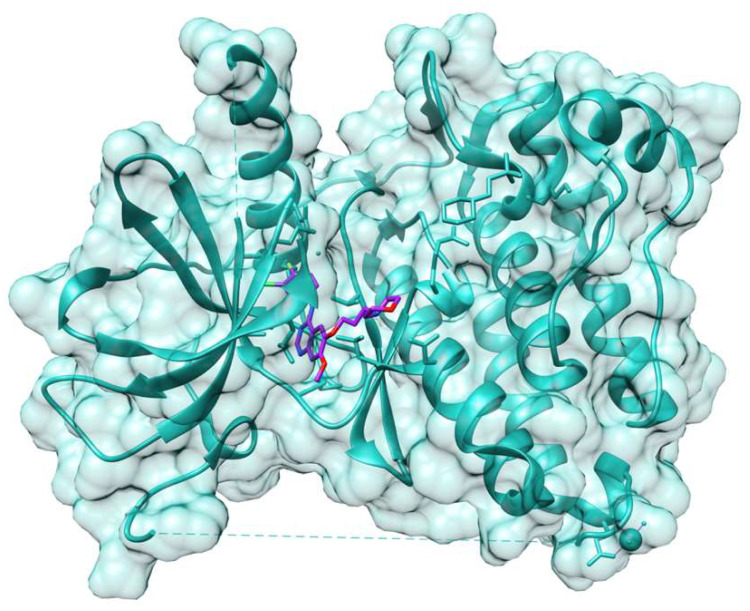
Gefitinib in complex with EGFR (PDB ID: 4WKQ; the image was obtained using UCSF Chimera, San Francisco, CA, USA).

The availability of large collections of molecules, the development of a large number of microscale analytical tools, the genome-wide association studies, and the simple and fast methods for the detection of target genes having a single-nucleotide polymorphisms took modern medicinal chemistry to the precision medicine era. The early steps of precision medicine were taken in the oncology field. The personalized therapies of the anticancer drugs, along with the identification of tumor-specific targets, were in part due to the general cytotoxicity and, as a consequence, the severe side effects of existing one-size-fits-all cancer drugs [9]. Examples are the molecularly targeted cancer therapies, such as small-molecule kinase inhibitors blocking the incorrect signaling of tumor cells from the intracellular side of a growth factor receptor protein, and monoclonal antibodies that often stop the same signal from outside the cell membrane. An early application of this was the epidermal growth factor receptor (EGFR), abnormally activated in cancer, against which the two classes of anti-EGFR agents, monoclonal antibodies and low-molecular-weight tyrosine kinase inhibitors, showed antitumor activity in patients. It was also reported that the kinase inhibitor gefitinib (Figure 4) and the monoclonal antibody cetuximab share complementary mechanisms of action on EGFR and that a combined EGFR targeting is a clinically exploitable strategy [54].

Many dysregulated pathways are now characterized, and new targets, proteins, and polynucleotides are attracting medicinal chemists. Among the new targets are not only the classical receptors, but many enzymes that can be inhibited by binding the small molecule to them, as in the case of the BCL-2 inhibitor venetoclax currently on the market [55], or by hitting a regulator protein [56]. To better understand the mechanism of action of drugs and to progress in the field of pharmacodynamics and precision medicine, we need to know the different conformations that the targets, proteins, or polynucleotides, assume in their energy minima during their functioning within the natural environment.

## 3. Pharmacokinetics

Pharmacokinetics, i.e., what is happening in the body to the drug molecule before and after the interaction with the target, is often divided in absorption, distribution, metabolism, and excretion (ADME). Many factors can influence the individual response to pharmaceutical compounds, among which genomic differences, gut microbiome, sex, nutrition, age, stress, and health status are included. They can impact drug absorption and distribution, the metabolic profile, with the drug–drug and drug–food interactions, and the toxicity in an individual. As for molecular design, computer simulations based on artificial intelligence help with the recognition of toxicity of the administered drug candidate. For example, an algorithm based on DL correctly predicted the toxicity of drug compounds in the data set with an accuracy of over 80% in almost all instances and was the Tox21 Data Challenge winner [33]; a similar approach was employed to study the possible epoxidation sites of drug candidates, obtaining a detailed picture on the likeliness of a molecule to be epoxidized and its consequent toxicity due to the structural modification [57]. On the experimental side, the advances made in gene sequencing, mainly using next-generation sequencing technologies [58] for pharmacogenomic studies and in the chemical analysis of metabolites, in particular by HPLC-MS, allows the better characterization the individuals and move toward what is commonly defined as precision medicine, not only as far as the target selection in a pathological state, but also for the pharmacokinetic effects. Precision medicine, which is defined as the capacity to prescribe the most effective treatment with the fewest adverse effects to a patient [59], applies principally to medical diagnostic, prescribing, and prevention [60], and is progressing very fast. A main problem to be resolved for precision medicine is the development of effective therapies targeted at the central nervous system (CNS). This is due to the failure to achieve therapeutically relevant concentrations in the CNS, due to the presence of the blood–brain barrier and to the strong neuronal interconnection between the different brain regions, as in the case of the dopaminergic effects of morphine and its derivatives targeted to the opioid receptors. A very important and challenging therapeutic area is that of brain tumors. Some of the approaches explored to address this challenge include blood–brain barrier disruption and drug modifications to enhance CNS permeability; unfortunately, neither approach has proven successful. Another approach is to deliver therapeutics locoregionally, directly into the tumor mass and the surrounding tumor-infiltrated brain parenchyma. The most widely used method for direct brain delivery is convection-enhanced delivery (CED), whereby specially designed catheters are introduced into target tissue, and the infusate is delivered slowly over a prolonged period of time. CED enables the delivery of conventional, nano-, bio-, gene, and even cellular therapies [61,62,63,64,65,66,67,68,69,70,71,72,73]. Hopefully, in the near future, it will be possible to deliver more small molecules in a therapeutic useful concentration and antibodies to the CNS.

Progress in precision medicine in the pharmacokinetic field is also increasing due to the improved use of experimental data on metabolic reactions and to the fact that the collection of DNA samples from clinical trial participants to perform pharmacogenomic studies has become standard practice for most pharmaceutical companies [74]. The analysis of single-nucleotide polymorphisms (SNPs) is rapidly growing, in particular for genetic variants that alter the activity of drug metabolizing enzymes and drug transporters.

**Figure 5:**
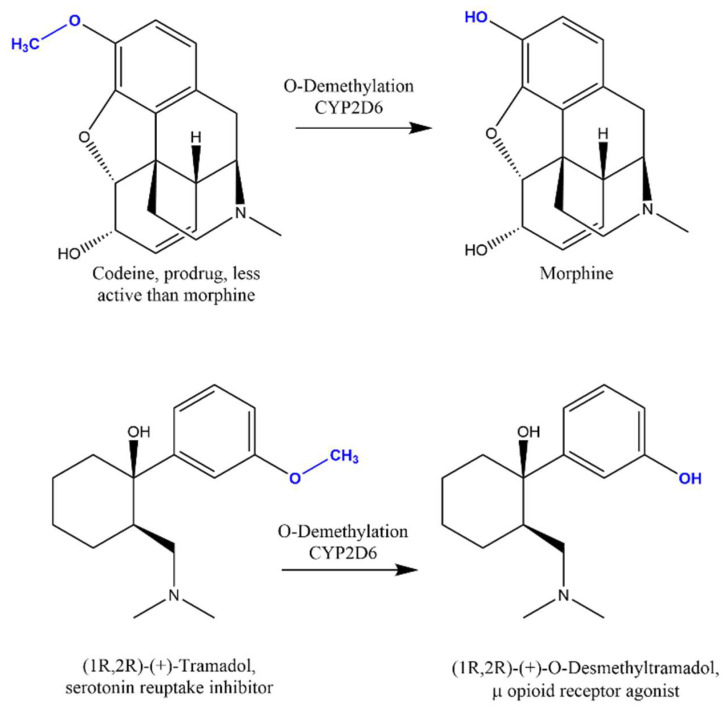
Examples of metabolic pathways for which CYP2D6 polymorphism is important.

As far as the experimental data is concerned, the massive work performed to gain information on metabolic pathways and the relative metabolizing enzymes of clinically used drugs to better understand their therapeutic effect is central to understanding the therapeutic drug properties, as well as the drug–drug and drug–food interactions. The metabolism of opioids, also considering their low clinical dosage, always attracted the attention of many investigators [75]. The developments in the pharmacokinetics of opioids is considered as a case study to briefly show the role of metabolism as a predictor of the clinical response and side effects of opioid analgesics, keeping the opioid crisis in mind [76]. The important side effects are due to the neuronal connectivity between the reward, dopaminergic, and opioid regions, as well as to the respiratory depression in the CNS, while many other side effects, e.g., constipation, are derived from the interaction with the peripheral opioid receptors. The common metabolic phase I reactions of opioids are dealkylations, O-dealkylation being CYP2D6-mediated, while N-dealkylation is CYP3A4-mediated, and redox reactions (e.g., for oxycodone and methadone); for phase II, glucuronation at positions three and six of the morphine nucleus and on reduced keto groups or dealkylated ethers, is the most important (Figure 5).

CYP2D6-mediated O-dealkylation of morphine 3-methoxy derivatives, such as codeine, and tramadol, are required to generate the phenolic OH group important for binding to a histidine of the opioid receptor. CYP2D6 is highly polymorphic, and the expression of different variants results in several phenotypes. The implementation of pharmacogenetics-based codeine prescribing that accounts for the CYP2D6 metabolizer status was described in a recent work [77] and is an example of precision medicine. Genome-wide association studies and candidate gene findings suggest that genetic approaches may help in choosing the most appropriate opioid and its dosage, while preventing adverse drug reactions [78].

Beyond the experimental data on metabolic enzymes and transporters, it is also possible to examine the genetic variants that alter the activity of enzymes or transporters and to use this information in ADME and toxicity studies [74,79]. Pharmacogenomic studies provide a growing list of clinically relevant markers that could be used to improve patient care [80], but such information is still not widely used in clinical practice. The difficulty of translating the pharmacogenomic information into ADME and toxicity studies during clinical phases was examined [81], but the basic reason is that the drug response is often highly complex, resulting from the interaction of many influencing factors. In the future, this approach will be a very useful tool for helping in the drug discovery process and in personalized medicine.

## 4. Conclusions

The maze of the drug discovery process is still very complex and challenging, even when only considering the small-molecule approach and no other promising approaches, such as those involving monoclonal antibodies or polynucleotides. Precision medicine, from drug discovery to the bedside, is the main concern nowadays. New powerful tools are made available almost every day, but medicinal chemists are still looking in every direction, from natural products [82] to sophisticated modeling [83], in search of new drug candidates complying with the new targets emerging from precision medicine needs. To further progress in the medicinal chemistry field, we need, in addition to new targets, a more accurate description of their different conformations and possibly of the evolution of the target structure with time during the biological process. This, combined with the knowledge of the genetic variants of the targets, will lead to an increased number and precision of the “magic bullets” that are drugs, and allow the progress of precision medicine.

## Data Availability

Not applicable.
